# Population Genetics of the Aquatic Fungus *Tetracladium marchalianum* over Space and Time

**DOI:** 10.1371/journal.pone.0015908

**Published:** 2011-01-14

**Authors:** Jennifer L. Anderson, Carol A. Shearer

**Affiliations:** Department of Plant Biology, University of Illinois at Urbana-Champaign, Urbana, Illinois, United States of America; University of Maribor, Slovenia

## Abstract

Aquatic hyphomycete fungi are fundamental mediators of energy flow and nutrient spiraling in rivers. These microscopic fungi are primarily dispersed in river currents, undergo substantial annual fluctuations in abundance, and reproduce either predominantly or exclusively asexually. These aspects of aquatic hyphomycete biology are expected to influence levels and distributions of genetic diversity over both spatial and temporal scales. In this study, we investigated the spatiotemporal distribution of genotypic diversity in the representative aquatic hyphomycete *Tetracladium marchalianum*. We sampled populations of this fungus from seven sites, three sites each in two rivers in Illinois, USA, and one site in a Wisconsin river, USA, and repeatedly sampled one population over two years to track population genetic parameters through two seasonal cycles. The resulting fungal isolates (N = 391) were genotyped at eight polymorphic microsatellite loci. In spite of seasonal reductions in the abundance of this species, genotypic diversity was consistently very high and allele frequencies remarkably stable over time. Likewise, genotypic diversity was very high at all sites. Genetic differentiation was only observed between the most distant rivers (∼450 km). Clear evidence that *T. marchalianum* reproduces sexually in nature was not observed. Additionally, we used phylogenetic analysis of partial *β*-tubulin gene sequences to confirm that the fungal isolates studied here represent a single species. These results suggest that populations of *T. marchalianum* may be very large and highly connected at local scales. We speculate that large population sizes and colonization of alternate substrates in both terrestrial and aquatic environments may effectively buffer the aquatic populations from in-stream population fluctuations and facilitate stability in allele frequencies over time. These data also suggest that overland dispersal is more important for structuring populations of *T. marchalianum* over geographic scales than expected.

## Introduction

Within-species genetic diversity is distributed spatially among populations and is variable over time. Levels and distributions of this diversity can be assessed using data from molecular markers (*e.g.* microsatellites) in conjunction with population genetic analyses. In the case of aquatic hyphomycete fungi, which are microscopic, submerged in water, and grow into and through opaque substrates, population genetic studies can generate unprecedented insight into otherwise unobservable biological phenomena.

Aquatic hyphomycetes are saprotrophic fungi that are fundamental mediators of energy flow and nutrient spiraling in stream food webs [1]–[4]. More than 90% of the carbon in wooded stream food webs originates from terrestrial vegetation [5], [6] but is initially inaccessible to aquatic invertebrates, which generally do not produce the enzymes necessary to digest recalcitrant plant compounds [7]. In the process of obtaining their own nutrition, aquatic hyphomycetes digest plant debris (*e.g.* [8], [9]), thereby promoting decomposition and making carbon and nutrients available to aquatic invertebrates [10], [11].

Deciduous leaves, the primary substrate of aquatic hyphomycetes, are highly seasonal in abundance in temperate climates [12]–[14], with leaves most abundant in late autumn and winter and least abundant in summer and early fall [15]. Similar seasonal fluctuations in aquatic hyphomycete abundances are also observed in temperate streams [15]–[18]. For example, sporulation rate [18], the fraction of leaves colonized by aquatic hyphomycetes [17] and within stream fungal biomass [15] decrease with substrate availability. Similar dynamics have been reported from streams around the world (*e.g.* [14], [19]–[26]) making these annual fluctuations one of the best-studied aspects of aquatic hyphomycete biology. The magnitude of these fluctuations is substantial, inspiring their description as “boom-bust” cycles [27].

In spite of their seasonal dynamics, populations of aquatic hyphomycetes are maintained in streams over time (*e.g.* [18], [22], [26], [28], [29]); however, the impact of the boom-bust cycles on levels of genetic diversity and the genetic structure of aquatic hyphomycete populations over time is unknown. One possibility is that these boom-bust cycles are the equivalent of annual population bottlenecks, which are expected to result in reduced genetic diversity and random changes in allele frequencies over time due to the genetic drift inherent in small populations [30]–[32]. However, previous studies have revealed high levels of genotypic diversity in populations of these fungi [33]–[36]. As single time point studies, however, they do not address the possibility of changes in genotypic diversity or population structure in populations of these fungi over time. To understand if/how fluctuations in fungal abundances impact genetic diversity and population structure in aquatic hyphomycetes, repeated sampling of populations must be undertaken.

Aquatic hyphomycetes are primarily dispersed in river currents as spores, either free-floating or trapped in foam. The asexually produced spores of these fungi are generally sigmoid, tetraradiate, or highly branched in shape, which may assist in their downstream dispersal [33]. There is evidence that spores can travel downstream in water as far as 1.8 km [37]. Some aquatic hyphomycetes can also survive passage through the digestive tracts of aquatic detritivores (*e.g.* [38]); therefore, aquatic animals may provide an additional means of within stream dispersal. It is unknown if or how animal mediated dispersal impacts aquatic hyphomycete population structure. Given that aquatic hyphomycete spores are primarily dispersed in river currents, and thus unidirectionally, genetic differentiation between rivers [35] and hierarchical population structure within individual rivers and watersheds could occur [39], [40]. Previous aquatic hyphomycete population genetic studies have sampled from only one stream [33], [34], or from one site in each of several streams in different drainages [35]; thus, we cannot yet determine whether or not aquatic hyphomycete distributions have a hierarchical component.

Alternative modes of dispersal, enabling dispersal across terrestrial barriers, must also occur to generate the broad, nearly cosmopolitan distributions observed for most species of aquatic hyphomycetes [41]. For example, *Tetracladium marchalianum* de Wild. occurs in temperate and sub-tropical areas worldwide [42], [43] including Hawaii, USA [44], which has never had a freshwater link to any other body of land. Overland dispersal could also explain the isolation of genotypically identical individuals of *Tetrachaetum elegans* Ingold from separate streams in the Montagne Noire region of France [35]. Although alternative modes of overland dispersal have been proposed for these fungi, including transportation by wind, insects, water droplets, waterfowl, and as hyphae in fragments of dry leaves [9], [41], [45], [46], it is not yet known which occur in nature. The frequency of overland dispersal is also unknown, but has important consequences for the geographic structuring of genetic diversity among populations of these fungi. For example, if overland dispersal of aquatic hyphomycetes is common, populations in neighboring streams may be genetically homogeneous and genetic structure within streams unlikely. Because aquatic hyphomycetes are microscopic and have few distinguishing/measurable morphological characters, dispersal of these fungi can best be inferred from species distributions and molecular population genetics studies.

Reproductive mode also has important implications for levels [47] and distributions of genetic diversity in populations of aquatic hyphomycetes, as well as the evolutionary potential of populations [48], [49]. Most aquatic hyphomycetes are only known to reproduce asexually; only 34 of about 300 aquatic hyphomycetes have known sexual states [50]. However, determination of which reproductive modes (sexual and/or asexual) are occurring in fungi is not straightforward. Fungi have different morphologies that accompany sexual and asexual reproduction and sexual states may be separated from asexual states by time, substrate, habitat, climate or other factors. For these reasons, mycologists have increasingly turned to molecular marker and DNA sequence data to determine whether fungi known only in an asexual state reproduce sexually in nature [51].

In this study, we use one of the most commonly occurring [43], [45], [52], [53] and distinctive [53]–[55] species of aquatic hyphomycete, *T. marchalianum*, as a model to track population genetic parameters over time, through population reduction and subsequent expansion, and over spatial scales ranging from within streams to between streams separated by 450 km, providing the first spatiotemporal population genetic analysis of an aquatic hyphomycete. Because *T. marchalianum* is only known to reproduce asexually but may undergo sexual reproduction in nature, we use multilocus genotype data to look for evidence of genetic recombination. Additionally, using partial *β*-tubulin sequence data we verify that morphologically identified isolates of this fungus represent a single species, insuring that analysis and interpretation of these data are not confounded by the presence of cryptic species (species that are morphologically indistinguishable).

## Methods

### Organism and Study Sites

*Tetracladium marchalianum* is only known to reproduce via mitosis, either through the production of distinctive tetraradiate haploid spores (Figure S1; see also [53]–[55]) or fragmentation of non-reproductive hyphae. The main axis of each spore (26–46 µm long) (measurements and terminology from [53]) has 1–3 septations with a globose distal cell (relative to the cell from which it is produced). Mature spores have three branches (25–40 µm×1.5–2.5 µm), one of which originates from the side of a shorter branch and terminates in a second central globose cell (combined size 31–41 µm×1.5–4 µm). *Tetracladium marchalianum* is known to degrade carboxymethylcellulose, xylan, and polygalacturonic acid [56], and its role in leaf decomposition in streams is well established [57]–[59]. Phylogenetic analyses indicate that within the Ascomycota, *T. marchalianum* belongs within the class Leotiomycetes and possibly within the order Helotiales [54]. The average genome size of taxa in this order is 1C = 0.03 pg, or approximately 27.14 Mb (Fungal Genome Size Database www.zbi.ee/fungal-genomesize, accessed on 6/26/2009).

**Figure 1:**
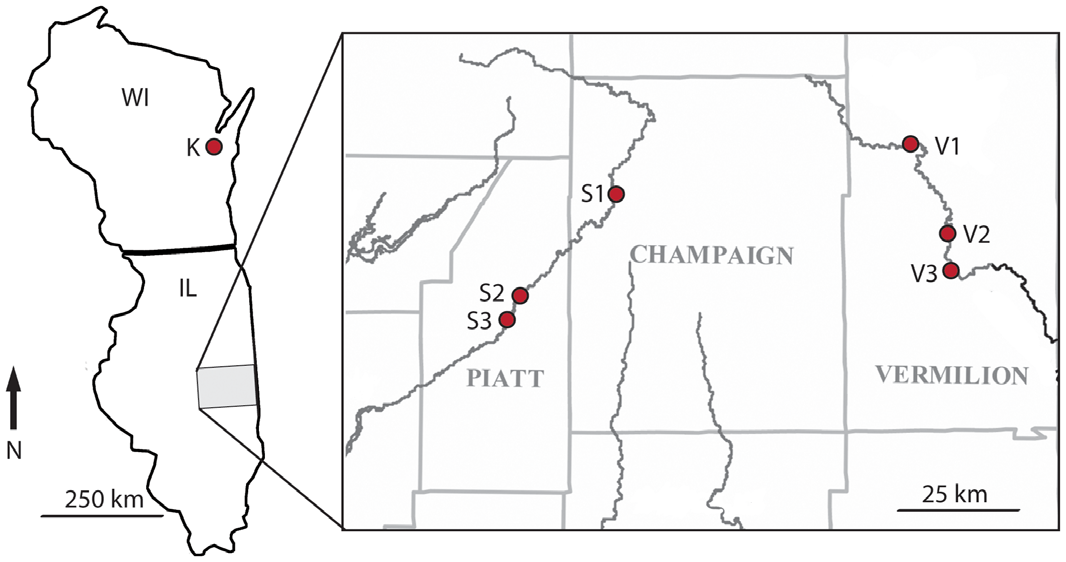
Collection sites in Wisconsin and Illinois, USA. Collections were made from three sites each in the Sangamon River (S1, S2, S3) and the Middle Fork of the Vermilion River (V1, V2, V3), and from one site in Konkapot Creek (K).

The three rivers sampled in this study were the Middle Fork of the Vermilion (Vermilion) and Sangamon Rivers in Illinois, USA, and Konkapot Creek in Wisconsin, USA (Figure 1). The Vermilion and Sangamon Rivers, which drain neighboring watersheds, were each sampled at three sites (Figure 1). In a downstream direction, the sites in the Vermilion River (V1, V2, V3) were separated by 7 and 9 km of channel length, and in the Sangamon River (S1, S2, S3) by 30 and 10 km of channel length. Konkapot Creek (K) is approximately 450 km north of the Illinois sites and only one site in Konkapot Creek was sampled. All sites were bordered by eastern deciduous forest with *Quercus* (oak) and *Acer* (maple) common at all sites, and *Platanus* (sycamore) also present at the Illinois sites.

### Sample collection and spore isolation

All seven sites were sampled in December 2002. Eight additional collections were taken from S1 between March 2002 and March 2004 (Table 1). No collections were made within two weeks following substantial, potentially riverbed scouring, rain events. For each collection 60–100 individual submerged leaves or leaf fragments from naturally occurring leaf packs, no more than one leaf per pack, were removed from the river, placed in individual plastic bags which were sealed and stored at 10°C until they were prepared for single spore isolation (1–5 days). Each leaf was rinsed with deionized water and 10–12 leaf discs (6 mm in diameter) were cut using a sterilized handheld paper hole puncher. The discs from an individual leaf were incubated together in deionized water in a Petri dish at 10°C under continuous fluorescent light. Dishes were examined weekly for sporulation using a dissecting microscope until *T. marchalianum* spores were observed, maximally one month. Individual spores were isolated from the dishes and allowed to germinate on antibiotic water agar [AWA: 1.8% Bacto Agar (BD Difco™, Franklin Lakes, NJ) plus 0.5 mg/ml Streptomycin Sulfate and Penicillin G (Sigma-Aldrich, St. Louis, MO) per 1 L deionized water] in Petri dishes. The identity of each germinating *T. marchalianum* spore was visually confirmed using a light microscope within 24 hours of plating on AWA. Individual spores were transferred to Potato Dextrose Agar with antibiotics [PDA+: 3.9% Potato Dextrose Agar (Difco) plus 0.5 mg/ml Streptomycin Sulfate and Penicillin G (Sigma) per 1 L deionized water] and grown at room temperature with ambient light. Up to thirty independent isolates from each collection (each originating from a different leaf) were randomly selected for DNA extraction and genotyping at 8 polymorphic microsatellite loci (GenBank accessions DQ272269—DQ272276) as previously described [36], [43].

**Table 1:** Collection site, sample, and colonization frequency details.

Site	ID	Date (m/d/y)	Latitude (N)	Longitude (W)	No. leaves	Fraction colonized
S1	Mar02	03/04/02	40°13.79′	88°22.59′	60	0.63
	Oct02	10/31/02			70	0.37
	Dec02/S1	12/20/02			59	0.51
	Mar03	03/02/03			100	0.72
	May03	05/04/03			100	0.17
	Jul03	07/06/03			100	0.20
	Aug03	08/29/03			100	0.03
	Oct03	10/25/03			100	0.58
	Dec03	12/18/03			100	0.63
	Mar04	03/05/04			100	0.44
S2	S2	12/17/02	40°4.13′	88°33.92′	74	0.59
S3	S3	12/17/02	39°59.96′	88°38.99′	75	0.55
V1	V1	12/13/02	40°13.60′	87°45.32′	75	0.49
V2	V2	12/13/02	40°9.14′	87°44.25′	75	0.53
V3	V3	12/13/02	40°6.92′	87°43.52′	75	0.47
K	K	12/31/02	44°14.65′	88°15.84′	100	0.47

Details of the 16 collections for this study including location, identifier (ID), date, the number of leaves collected, and the fraction of leaves colonized by *T. marchalianum*. For site abbreviations refer to Figure 1. Note that the collection from S1 in December 2002 is used in both spatial and temporal analyses and referred to as Dec02 or S1 accordingly.

### Data analysis

Genotypic diversity [60], the probability of two individuals sampled at random from a population having different genotypes, was calculated using MultiLocus 1.3b [61]. Individuals with identical multilocus genotypes were identified using a custom Python script (individuals with missing data were excluded). Allelic richness was calculated using CONTRIB [62] with rarefaction to the smallest sample size obtained minus 1 to produce estimates that are unbiased by differences in sample sizes.

Genetic differentiation was assessed using Weir and Cockerham's [63] estimator of *F_ST_*, *theta θ*, in FSTAT v 2.9.3.2 [64]. Because isolates of *T. marchalianum* were taken from different types of leaves (oak, maple, sycamore, or unidentified) genetic differentiation by leaf type was also analyzed to ascertain if genotype–substrate specificities influence genetic structure in this species. Genetic structure was also evaluated using a Bayesian approach in Structure v2.3.2 [65]–[67] for K = 1 to K = 7 with 100,000 steps following a 50,000 step burn in. Both admixture models (present/absent) were employed, allele frequencies were assumed to be independent, and sample origin information was disregarded.

The phylogenetic relationships among 19 *T. marchalianum* isolates (18 from this study and 1 from Europe), 2 sister species (*T. setigerum* and *T. maxilliforme*), and 4 additional species from within the Order Helotiales [*Rhynchosporium secalis* (GenBank Accession X81046.1), *Sclerotinia sclerotiorum* (AY312374.1), *Botryotinia fuckeliana* (X73133.1), and *Monilinia fructicola* (AY283678.1)] were determined from partial *β*-tubulin gene sequences. Uncorrected sequence divergence percentages between *Tetracladium* individuals were also generated. All *Tetracladium* sequences (GenBank accessions HQ123547—HQ123567) were obtained using primers BT1819R and BT2916 [68]. The Maximum Likelihood (ML) tree, based on 889 unambiguously aligned nucleotides, was estimated using PhyML v2.4.5 [69]. A GTR+I+G model of nucleotide evolution was assumed with all parameters of the model estimated during the analysis. Support for each branch was determined as the minimum of the Chi^2^-based and SH-like support values; values greater than 0.90 indicate significant support for a node.

Multilocus linkage disequilibrium was evaluated using the Index of Association, *I_A_*, where if *I_A_* of the data is significantly different from the results of 1000 randomized datasets (p≤0.05) the null hypothesis of recombination is rejected. The value of *I_A_* is known to increase with the number of loci tested; therefore, multilocus linkage disequilibrium was also measured using the locus number independent measure *r_D_*, [61]. Both *I_A_* and *r_D_* were calculated in MultiLocus 1.1.3b [61]. Important limitations of analyses to determine reproductive mode should be noted. Asexual population structure can result from selfing (homothallic sexual reproduction), clonality, or sexual reproduction in populations that are genotypically homogeneous: analyses of reproductive mode based on genotypic diversity alone cannot distinguish among these factors if asexual population structure is observed.

## Results

### Collections

*Tetracladium marchalianum* was sampled over both spatial (S1, S2, S3, V1, V2, V3, K) and temporal (S1: Mar02–Mar04) scales. Note that the December 2002 collection from S1 is included in both spatial and temporal spatial comparisons and is referred to as S1 and Dec02 accordingly. The 16 collections undertaken for this study (Table 1) resulted in the sampling of 1363 leaves and isolation of 1036 cultures of *T. marchalianum*, each originating from a single asexually produced spore of verified morphological identity. The Aug03 collection yielded only three single spore isolates and has been excluded from the genetic analyses presented below due to small sample size. The relative proportions of maple, oak, sycamore, and unknown leaf types in each collection varied (Figure S2 A, B). This variation was largely mirrored by the relative proportions of leaves of each type that were colonized by *T. marchalianum* (Figure S2 C, D). Collections from S1 in 2002 were made before (Mar02) and after (Oct02) the population reduction occurred in that year. The sampling scheme was adjusted to span the event the following year.

### Population over time

The fraction of leaves colonized by *T. marchalianum* at S1 was highest fall through spring (0.37–0.72) and smallest in summer (0.03–0.20) (Table 1). From March to August 2003 colonization frequency declined by 69%, with 80% of the reduction occurring between March and May. This reduction was followed by a substantial increase in colonization frequency in fall 2003. Inter-annual variation in colonization frequency was also observed (*e.g.* Mar02 = 0.63, Mar03 = 0.72, Mar04 = 0.44) (Table 1).

### Diversity

Multilocus genotypes were obtained for 211 individuals from S1, collected between March 2002 and 2004, and 180 individuals from the 6 additional sites in Illinois and Wisconsin. In some cases fewer than 30 individuals per collection were genotyped due to the exclusion of samples that yielded low DNA concentrations and/or the isolation of fewer than 30 independent isolates (Table 2). We observed 3 to 18 alleles per marker, with an average of 4.77 alleles per locus per sample (range 4.13–5.5; Table 2). After rarefaction, the average allelic richness among all loci and populations was 3.14 (2.60–3.53; Table 2).

**Table 2:** Measures associated with diversity, population structure, and mode of reproduction in *T. marchalianum*.

ID	N	No. types	Max identical	Genotypic diversity	Alleles per locus	Allelic richness	Fraction Group 1	*I_A_*	*r_D_*
Mar02	26	22	3	0.982	4.38	2.984	0.65	1.644*	0.236*
Oct02	24	23	1	0.996	4.88	3.413	0.46	1.042*	0.152*
Dec02	30	29	2	0.998	5.25	3.482	0.67	1.281*	0.185*
Mar03	30	24	3	0.982	4.75	3.015	0.53	1.531*	0.221*
May03	17	16	2	0.993	4.50	3.426	0.76	0.874*	0.125*
Jul03	19	18	2	0.994	4.38	3.243	0.74	1.412*	0.204*
Oct03	30	29	1	0.998	5.38	3.421	0.60	1.002*	0.145*
Dec03	30	23	2	0.966	4.75	2.965	0.50	1.387*	0.198*
Mar04	30	27	3	0.984	4.38	2.789	0.67	1.213*	0.176*
S2	30	22	3	0.961	4.13	2.651	0.50	1.888*	0.271*
S3	30	26	3	0.989	5.38	3.366	0.38	1.454*	0.210*
V1	30	28	1	0.995	4.63	3.149	0.63	0.625*	0.090*
V2	30	26	3	0.990	4.75	3.033	0.43	1.601*	0.231*
V3	30	28	2	0.998	5.50	3.533	0.57	1.129*	0.163*
K	30	25	3	0.986	4.50	2.598	0.97	0.502*	0.073*

The number of individuals genotyped (N) from each collection and site, number of distinct multilocus genotypes observed (No. types), maximum number of identical individuals (excluding individuals with missing data), Nei's genotypic diversity, mean number of alleles per locus, allelic richness with rarefaction to 16, the fraction of each population identified as belonging to Group 1 in Structure, Index of Association (*I_A_*), and a locus number independent measure of multilocus linkage disequilibrium (*r_D_*).

*p<0.001.

Genotypic diversity was very high in all collections (0.961–0.998; Table 2), regardless of time and site, and no associations between season or colonization frequency and genotypic diversity were observed. Nearly every individual in each collection had a unique multilocus genotype; however, we observed up to 3 identical individuals per collection (Table 2). Identical individuals were also observed in collections separated by both space and time, with one genotype observed in 14 individuals over two years, and 3 genotypes were common to 5 sites.

### Differentiation

Population differentiation, based on pairwise comparisons of *F_ST_*, was observed over both spatial (Table 3) and temporal scales (Table 4). Spatially, population structure was only observed at the largest scale tested, between streams separated by ∼450 km, with no differentiation observed within streams or between streams in neighboring watersheds. Temporally, moderate differentiation, 0.05–0.15 [70] with statistical support was observed in four comparisons (Table 3), and indicates differences between the Dec03 and Mar04 collections and 4 collections in or before July 2003. Analyses of genetic differentiation by leaf type do not indicate that substrate-specificity influences population structure in *T. marchalianum* (Tables S1 and S2).

**Table 3:** Pairwise comparisons of *F_ST_* between collections of *T. marchalianum* from Wisconsin and Illinois, USA.

	S1	S2	S3	V1	V2	V3	K
S1		0.010	0.067	0.002	0.057	-	0.097*
S2	0.205		0.007	-	0.005	-	0.200*
S3	0.014	0.150		0.017	-	0.003	0.267*
V1	0.383	-	0.138		0.009	-	0.149*
V2	0.005	0.081	-	0.157		-	0.248*
V3	-	-	0.500	-	-		0.169*
K	0.002*	0.002*	0.002*	0.002*	0.002*	0.002*	

*F_ST_* (upper diagonal matrix). Negative *F_ST_* values are excluded (-). P-values obtained after Bonferroni corrections (lower diagonal matrix).

*significant at the 5% nominal level.

**Table 4:** Pairwise comparisons of *F_ST_* between collections of *T. marchalianum* from S1 between March 2002 and March 2004.

	Mar02	Oct02	Dec02	Mar03	May03	Jul03	Oct03	Dec03	Mar04
Mar02		-	0.009	0.001	0.019	0.042	-	0.016	0.001
Oct02	-		0.012	-	0.032	0.048	-	0.038	0.069
Dec02	0.140	0.208		0.012	-	-	0.010	0.078*	0.058
Mar03	0.097	-	0.168		0.035	0.045	-	0.037	0.066*
May03	0.078	0.047	-	0.024		0.001	0.035	0.111*	0.093
Jul03	0.018	0.071	-	0.068	0.167		0.028	0.135	0.103*
Oct03	-	-	0.231	-	0.018	0.107		0.021	0.027
Dec03	0.011	0.006	0.002*	0.003	0.001*	0.003	0.057		0.009
Mar04	0.147	0.004	0.007	0.001*	0.003	0.001*	0.026	0.294	

*F_ST_* (upper diagonal matrix). Negative *F_ST_* values are excluded (-). P-values obtained after Bonferroni corrections (lower diagonal matrix).

*significant at the 5% nominal level.

Genetic structure was also evaluated using Bayesian analyses as implemented by the software Structure. These analyses support the existence of two groups (K = 2) for both spatial and temporal data and both models tested. Both groups identified by Structure (Group 1 and Group 2) were represented in every collection, but in different frequencies (Table 2). Among collections from Illinois, an average of 58% (range 38%–76%) of individuals were identified as belonging to Group 1. The Wisconsin population was nearly entirely composed of Group 1 individuals (97%). Analyses of population differentiation (as above) by group, which yielded significant results only for Group 1 (Table S3), are qualitatively similar to the non-partitioned results and do not change the interpretation of these data. Note that partitioning the individuals between groups resulted in small per collection sample sizes.

### Cryptic species

**Figure 2:**
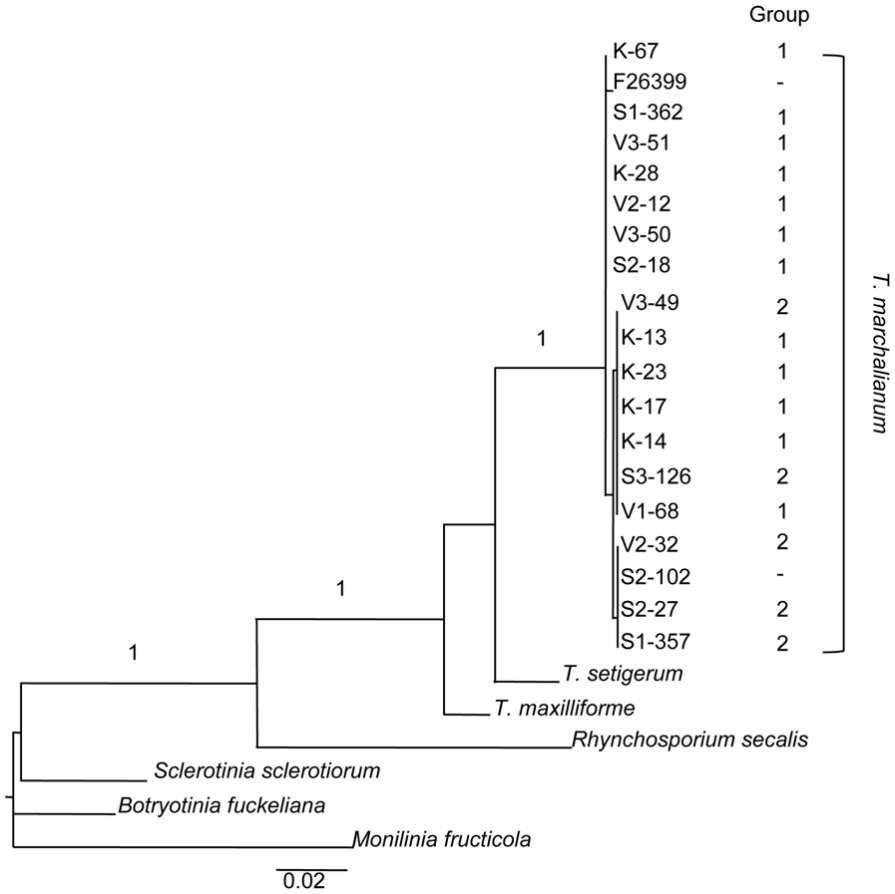
Maximum Likelihood tree for 19 isolates of *T. marchalianum* based on partial *β*-tubulin gene sequences. The tree resulting from ML analysis of partial *β*-tubulin gene sequences (889 unambiguously aligned nucleotides). Support for each branch is shown as the minimum of the Chi^2^-based and SH-like support values; only values greater than 0.90, indicating significant support for a node, are reported. The scale bar indicates the number of nucleotide substitutions per site. The group to which isolates of *T. marchalianum* were assigned by Structure is identified when known.

Based on Maximum Likelihood analysis of partial *β*-tubulin sequence data, isolates identified as *T. marchalianum* based on morphology represent a single species (Figure 2); there is no evidence for genetic structure among 19 *T. marchalianum* isolates. Furthermore, individuals identified as Group 1 or 2 in Structure are intermixed in the resulting phylogeny. Average sequence divergence among the 18 *T. marchalianum* individuals from Wisconsin and Illinois is very low (0.24%, range 0.00%–0.45%), and is an order of magnitude lower than that observed between *T. marchalianum* and its sister species *T. setigerum* (4.88%) and *T. maxilliforme* (5.52%). These data do not support the existence of cryptic species within *T. marchalianum*.

### Recombination

Reproduction in *T. marchalianum* appears to be primarily or effectively asexual (*i.e.* if sexual reproduction does occur, it has no discernable impact on linkage disequilibrium among the 8 loci employed in this study); the null hypothesis of recombination was rejected for all collections by the Index of Association (*I_A_* and *r_D_*; Table 2). Because the presence of multiple genotypically identical individuals could bias this metric, clone-corrected datasets were also analyzed (data not shown). Qualitatively similar results are obtained from both clone-corrected and uncorrected data, possibly due to the low frequency of identical individuals in these samples. However, when the partitioned (Group1 only) data are analyzed (Table S4) linkage equilibrium is not rejected for the sample from Konkapot Creek, nor from the Vermilion River (V1, V2, and V3 Group 1 individuals were combined due to small sample size).

## Discussion

Aquatic hyphomycetes primarily colonize autumn-shed leaves, a substrate that is inherently seasonal in abundance. The abundance of aquatic hyphomycetes in streams fluctuates seasonally in conjunction with the availability of autumn-shed leaves. It was previously unknown whether these contemporaneous fluctuations influence levels of genetic diversity or genetic stability over time in populations of aquatic hyphomycetes. In this study, we repeatedly sampled the population of *T. marchalianum* from leaf litter at one site (S1) in the Sangamon River, Illinois, allowing us to simultaneously track changes in the fraction of leaves colonized by this species, and the frequencies of microsatellite alleles and multilocus genotypes. The fraction of leaves colonized by *T. marchalianum* increased and decreased seasonally as expected (Table 1). Inter-annual variation in colonization frequency and the magnitude of the intra-annual changes we observed are comparable with previous reports for this species and consistent with observed fluctuations in spore density [17], [20], [71]. Colonization frequencies appear to be independent of leaf-type and variation in the relative proportions of leaf-types collected (Figure S2, Table 1). Additionally, leaf-type does not influence genetic population structure in this species (Tables S1 and S2), which is consistent with findings for another aquatic hyphomycete, *T. elegans* [35].

Although the annual reduction in fungal abundance in 2003 was substantial, a decline of more than an order of magnitude, we did not observe corresponding changes in genotypic diversity or allelic richness in the S1 population over time (Table 2). Rather, genotypic diversity was very high throughout the study in all collections and most of the individuals sampled had unique multilocus genotypes (Table 2). This result is consistent with previous single time-point reports for other aquatic hyphomycetes both with [33] and without known sexual states [34], [35]. For example, Laitung and colleagues [35] observed different AFLP genotypes in 83% to 100% of all isolates of *T. elegans* collected from nine streams in France.

From these data, it is clear that allele frequencies in populations of *T. marchalianum* can remain stable in spite of dramatic demographic fluctuations. Although there is evidence of moderate differentiation (0.05–0.15 [70]) between collections that bracket late summer/early autumn 2003 (Table 4), suggesting some variability in genetic structure in *T. marchalianum* over time, it is not clear that seasonal or substrate dynamics underlie this variation. A similar pattern of differentiation was not observed among collections spanning the summer of 2002. Furthermore, the lack of differentiation between March collections over three years (2002, 2003, 2004) illustrates that the population is remarkably stable over longer time scales. The outstanding question then is how can this genetic stability be achieved?

To our knowledge, direct estimates of aquatic hyphomycete population sizes, census sizes as opposed to relative measures, do not exist in the literature. However, if one considers that the late summer standing crop of coarse particulate organic matter (CPOM), which includes leaf litter, in North American headwater streams is about 20g C m^−2^ (ash-free dry mass [14]) and the average wet mass per intact leaf is approximately 1g (determined from autumn-shed oak and maple leaves submersed in water for 1 week) it is obvious that even when leaves are relatively least abundant, the number of leaves or leaf fragments remaining in streams could be very high and support large populations of *T. marchalianum*. This is especially true given that multiple genetically distinct aquatic hyphomycete individuals can occupy a single leaf [34]. As such, even 3% survival through a population decline could leave a considerable number of genotypically diverse individuals for population recovery the following year, facilitating the maintenance of high levels of diversity and preventing population differentiation over time.

The colonization of alternative substrates on land and in water might also foster the maintenance of genetic diversity and stabilize allele frequencies over time [72]. Aquatic hyphomycetes are known to colonize a variety of substrates including wood, which might serve as an important long-term source of fungal inoculum due to slower decomposition rates [9], [73]. Aquatic hyphomycetes are also known to have at least limited survival in substrates under dry conditions [74]–[78] and there is a growing understanding that some aquatic hyphomycetes function as saprotrophs in the aquatic environment and as endopyhtes in the terrestrial environment [72], [79], [80]. The introduction of fungal inoculum into streams from dried leaves or other colonized plant material could reduce the influence of fluctuating in-stream leaf litter availability on local genotypic diversity.

Aquatic hyphomycetes are primarily dispersed as spores in river currents; however, this mode of dispersal does not explain the global distributions of many aquatic hyphomycetes, including *T. marchalianum*. Thus, dispersal by alternative modes must occur. Neither the importance of alternative modes of dispersal, nor the frequency of gene flow between populations of aquatic hyphomycetes is currently known. Although unidirectional dispersal and gene flow can generate hierarchical population structure within and between rivers [39], [40], we do not observe this pattern. Rather, the collection of genotypically identical isolates of *T. marchalianum* from streams in neighboring and distant watersheds can be interpreted as evidence of overland gene flow (see also [35]). Furthermore, we did not observe genetic differentiation between any collections from the Sangamon and Vermilion Rivers (Table 3), even though collection sites were separated by up to 30 km within a single stream and by more than 60 km between streams (Figure 1). In contrast, differentiation between populations in neighboring streams has been reported for *T. elegans* in the more topographically diverse Montagne Noire region of France [35]. The lack of population differentiation within and between streams in Illinois suggests that regional gene flow substantially influences the structuring of genetic diversity among these populations of *T. marchalianum*.

Although we did not observe genetic differentiation between populations of *T. marchalianum* in Illinois, we did observe differentiation between the population of *T. marchalianum* in Konkapot Creek, Wisconsin and the Illinois populations (Table 3). Unlike the Illinois populations, the Konkapot Creek population appears to be composed almost entirely of individuals belonging to Group 1, as identified by Structure (Table 2). Note that individuals from Group 1 and Group 2 are intermixed in the phylogeny resulting from analysis of partial *β*-tubulin sequence data (Figure 2); thus, these groups do not signal the existence of cryptic species within *T. marchalianum*. Overall, we find no evidence of cryptic species in *T. marchalianum*; this finding is supported by published phylogenies based on 18S and internal transcribed spacer (ITS) rDNA [54], [81].

The results of this study, in combination with the work of Laitung and colleagues [35], suggest that gene flow frequencies for aquatic hyphomycetes are governed by extrinsic factors such as distance between streams and physical barriers to dispersal. By this reasoning, we might predict that isolation by distance maintains the genetic differentiation we observed between Konkapot Creek and the Illinois sites. It is also possible that the population in Konkapot Creek was founded from a more southern population following the retreat of the Laurentide Ice Sheet at the end of the last ice age. Additional studies of a phylogeographic nature will be necessary to identify and interpret the geographic distribution of genetic diversity in *T. marchalianum* and other aquatic hyphomycetes.

Fungi known only in an asexual state are generally assumed to have a corresponding sexual state. In this study, statistically significant results for *I_A_* suggest that *T. marchalianum* is effectively asexual (Table 2). The basis of this metric is linkage disequilibrium (the non-random association of loci), which is expected in asexually reproducing organisms, and breaks down quickly in response to recombination [47], [82]. However, high genotypic diversity is frequently interpreted as an indication of sexual recombination (*e.g.* [83], [84]). For *T. marchalianum*, high genotypic diversity, the relatively rare observation of individuals with identical multilocus genotypes (Table 2), and the non-significant results for *I_A_* in two populations when only Group 1 individuals are analyzed (Table S4) could suggest that this species undergoes sexual reproduction.

Analyses of reproductive mode, such as those performed in this research, commonly indicate that species do recombine in nature [83]. Ideally, consensus among multiple metrics occurs and is used to infer reproductive mode [51]. However, conflicting results are not uncommon nor is resolution of these conflicts standardized [35], [84], [85]. Unfortunately, both linkage disequilibrium and high genotypic diversity can result from processes unrelated to mode of reproduction, such as physical linkage, genetic drift, and population admixture in the case of linkage disequilibrium, and large population size, establishment of a population by a diverse founder population, immigration [86], somatic recombination [47] and mutation in the case of genotypic diversity. It is unlikely that physical linkage can explain the results of *I_A_* in this study because qualitatively similar results are also obtained from analyses based on 57 AFLP loci [43], which should provide coverage over the entire genome (the locations of the SSR markers in the *T. marchalianum* genome are as yet unknown). Furthermore, large population sizes (inferred from sampling success), lack of population differentiation over time or among sites, and evidence that cryptic species were not sampled in this study make related explanations for linkage disequilibrium in *T. marchalianum* unlikely. However, it remains difficult to decide whether high genotypic diversity is sufficient evidence to override the weight of the *I_A_* results presented here which indicate that *T. marchalianum* is effectively asexual in these populations.

There is a growing trend to use the distributions, presence/absence, and sequence variation of mating types and mating type alleles as evidence in determining reproductive mode [87]–[91]; research along these lines would complement the work presented here. This information could explain the apparent asexuality of the populations we sampled (for example, only one mating type may be present), or suggest that asexual reproduction is dominant at broader geographic scales if both mating types are co-occurring but no evidence of recombination is observed. It is also possible that *T. marchalianum* reproduces sexually in only some populations; different modes of reproduction can dominate different parts of a species range [92], [93].

### Conclusions

Seasonal fluctuations in the abundance of autumn-shed leaves and the corresponding changes in aquatic hyphomycete abundance are doubtlessly ecologically important for energy flow and nutrient spiraling in streams. However, these dynamics do not appear to influence the genetic make-up of *T. marchalianum* populations. The work presented here clearly shows that *T. marchalianum* populations can maintain high genotypic diversity and stable genetic structure regardless of annual boom-bust cycles. Both temporal stability and high genotypic diversity could indicate that populations of this fungus are very large, ensuring yearly population recovery and the maintenance of many genetically different lineages over time. Alternative habitats such as the roots and aerial leaves of living plants and woody debris in streams are also potentially important for the maintenance of diversity in populations of *T. marchalianum* and should be investigated further. Spatially hierarchical sampling of *T. marchalianum* populations revealed a lack of genetic differentiation at all but the largest scale (streams separated by ∼450 km), suggesting that overland dispersal and gene flow may be important drivers of diversity and genetic structure in this fungus, homogenizing populations within and between streams regionally. Thus, although the passive downstream dispersal of spores is the primary mode of aquatic hyphomycete dispersal, alternative modes of dispersal and their frequencies require attention. High genotypic diversity could indicate that *T. marchalianum* reproduces sexually in nature; however, genotypic diversity can be influenced by factors other than mode of reproduction. Thus it is not clear that high genotypic diversity should outweigh the results of analyses based on linkage disequilibrium, which indicate that this species is effectively asexual. We suggest that additional studies, including determination of the presence/absence, distribution, and sequence variation of mating types and mating type alleles, be undertaken before firm conclusions about the reproductive mode(s) employed by *T. marchalianum* are made.

## Supporting Information

Figure S1 **The asexually produced spore of** ***T. marchalianum*** **.** Three branches radiate from the central axis, one originates from the side of a shorter branch (right). Both this short branch and the central axis terminate in globose cells. This photograph was taken using Nomarski optics, measure bar = 10µm.
(TIF)

Figure S2 **The relative proportions of leaf types sampled in each collection (A, B) and colonized by** ***T. marchalianum*** **(C, D).** Leaf data is not available for the Oct03 collection. In Aug 03 only three isolates were obtained.
(TIF)

Table S1 (PDF)

Table S2 (PDF)

Table S3 (PDF)

Table S4 (PDF)
