# Supplementary figures and images for: Population Genetics of the Aquatic Fungus Tetracladium marchalianum over Space and Time
Source: PLoS One. 2011 Jan 14;6(1):e15908. doi: 10.1371/journal.pone.0015908 (PMC3021519; doi:10.1371/journal.pone.0015908)

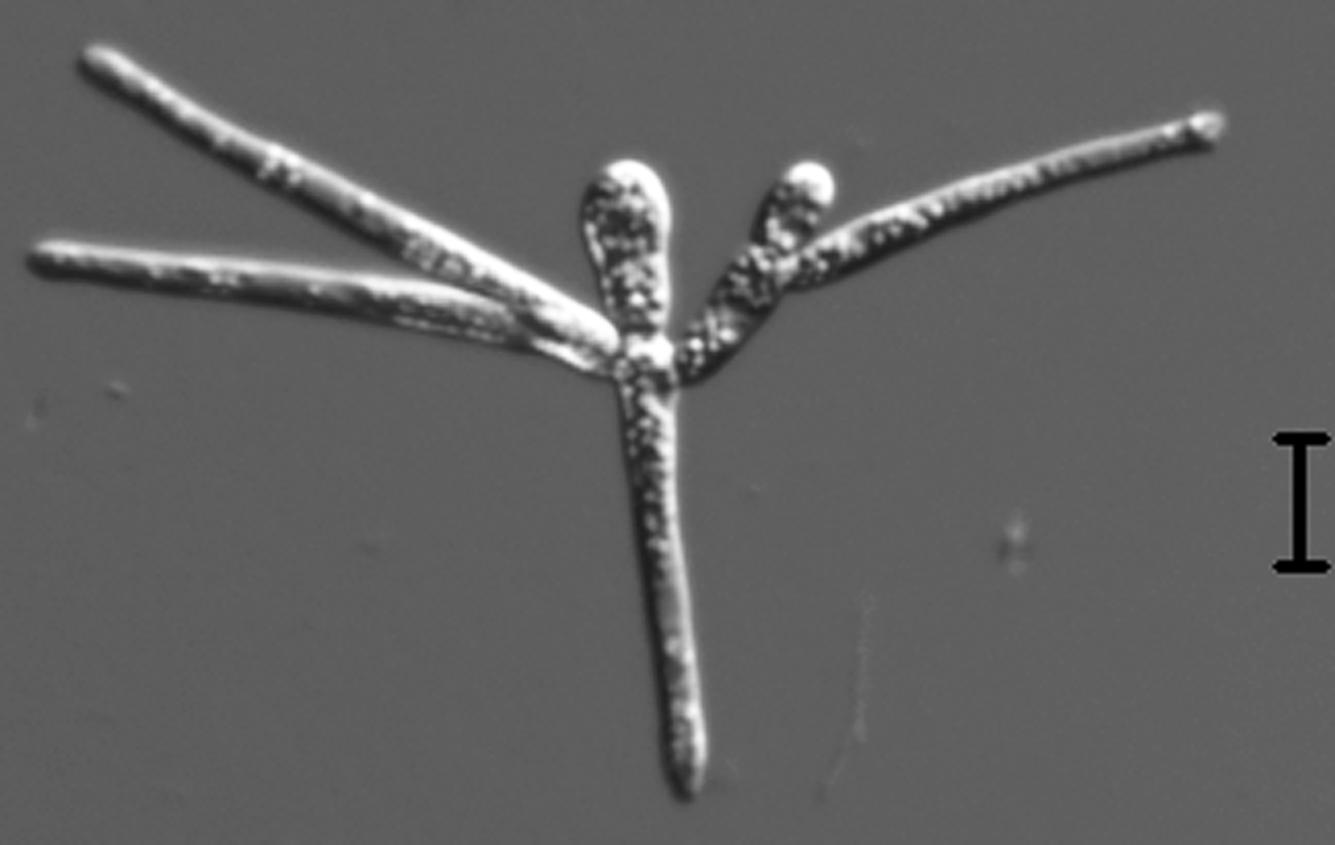

Supplement: Figure S1 — The asexually produced spore of T. marchalianum . Three branches radiate from the central axis, one originates from the side of a shorter branch (right). Both this short branch and the central axis terminate in globose cells. This photograph was taken using Nomarski optics, measure bar = 10µm. (TIF) [file pone.0015908.s001.tif]

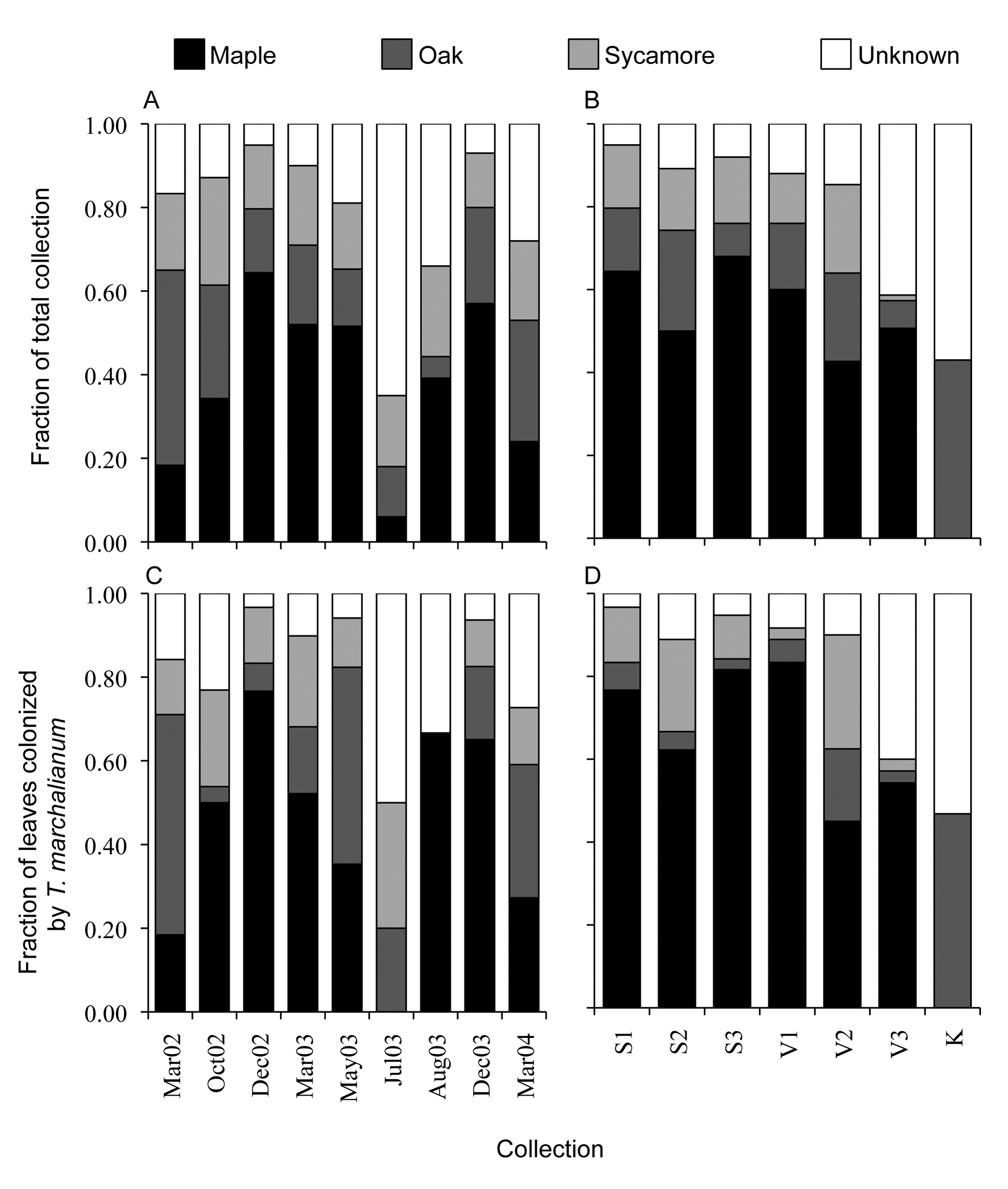

Supplement: Figure S2 — The relative proportions of leaf types sampled in each collection (A, B) and colonized by T. marchalianum (C, D). Leaf data is not available for the Oct03 collection. In Aug 03 only three isolates were obtained. (TIF) [file pone.0015908.s002.tif]
