# Supplementary material for: Population Genetics of the Aquatic Fungus Tetracladium marchalianum over Space and Time
Source: PLoS One. 2011 Jan 14;6(1):e15908. doi: 10.1371/journal.pone.0015908 (PMC3021519; doi:10.1371/journal.pone.0015908)
Supplement: Table S1 — (PDF) [file pone.0015908.s003.pdf]

Table S1 Pairwise comparisons of F_{ST} by substrate type for isolates of *T. marchalianum* collected at S1 between March 2002 and March 2004.

	Maple	Oak	Sycamore	Unknown
Maple		-	0.028	0.016
Oak	0.475		0.140	0.120
Sycamore	0.033	0.450		-
Unknown	0.183	0.183	0.867	

F_{ST} (upper diagonal matrix). Negative F_{ST} values are excluded (-). P-values obtained after Bonferroni corrections (lower diagonal matrix). No comparisons were significant at the 5% nominal level.
