# Supplementary material for: Population Genetics of the Aquatic Fungus Tetracladium marchalianum over Space and Time
Source: PLoS One. 2011 Jan 14;6(1):e15908. doi: 10.1371/journal.pone.0015908 (PMC3021519; doi:10.1371/journal.pone.0015908)
Supplement: Table S2 — (PDF) [file pone.0015908.s004.pdf]

Table S2 Pairwise comparisons of F_{ST} by substrate type for isolates of *T. marchalianum* collected in Illinois (V1, V2, V3, S1, S2, S3) in December 2002.

	Maple	Oak	Sycamore	Unknown
Maple		-	-	0.014
Oak	0.175		-	-
Sycamore	0.492	0.133		0.128
Unknown	0.167	0.725	0.208	

F_{ST} (upper diagonal matrix). Negative F_{ST} values are excluded (-). P-values obtained after Bonferroni corrections (lower diagonal matrix). No comparisons were significant at the 5% nominal level.
