# Supplementary material for: Population Genetics of the Aquatic Fungus Tetracladium marchalianum over Space and Time
Source: PLoS One. 2011 Jan 14;6(1):e15908. doi: 10.1371/journal.pone.0015908 (PMC3021519; doi:10.1371/journal.pone.0015908)
Supplement: Table S3 — (PDF) [file pone.0015908.s005.pdf]

Table S3 Pairwise comparisons of F_{ST} between collections using isolates designated by Structure as belonging to Group 1.

Comparison		F_{ST}
Oct02	Mar04	0.174
Dec02	Mar04	0.160
Mar03	Dec03	0.149
Mar03	Mar04	0.168
May03	Dec03	0.173
May03	Mar04	0.200
Jul03	Dec03	0.200
Jul03	Mar04	0.208
V2	K	0.139
V3	K	0.123
S2	K	0.141

Only values significant at the 5% nominal level after Bonferroni corrections are reported. Note, partitioning the data in this way resulted in small sample sizes for each group in each collection.
