# Supplementary material for: Population Genetics of the Aquatic Fungus Tetracladium marchalianum over Space and Time
Source: PLoS One. 2011 Jan 14;6(1):e15908. doi: 10.1371/journal.pone.0015908 (PMC3021519; doi:10.1371/journal.pone.0015908)
Supplement: Table S4 — (PDF) [file pone.0015908.s006.pdf]

Table S4 Analyses pertaining to mode of reproduction using data partitioned into Groups 1 and 2 as determined by Structure.

	**Group 1**		**Group 2**	
**Unit analyzed**	I_A	r_D	I_A	r_D
S1 series	0.194*	0.028*	0.446*	0.067*
Sangamon	0.326*	0.048*	0.542*	0.081*
Vermilion	0.043	0.006	0.707*	0.104*
Konkapot	0.190	0.028	-	-

Partitioning these data into the groups identified by Structure results in problems with small sample sizes. To minimize this problem the collections were grouped by site (S1 series contains all 10 collections from S1) and river [Sangamon (S1, S2, S3), Vermilion (V1, V2, V3)] for these analyses. The collection from Konkapot Creek was almost entirely designated as Group 1; therefore, analysis of Group 2 was not possible. $*p < 0.001$.
